# MicroRNA-1291 Is Associated With Locoregional Metastases in Patients With Early-Stage Breast Cancer

**DOI:** 10.3389/fgene.2020.562114

**Published:** 2020-12-02

**Authors:** Daniel Escuin, Laura López-Vilaró, Olga Bell, Josefina Mora, Antonio Moral, José Ignacio Pérez, Cristina Arqueros, Teresa Ramón y Cajal, Enrique Lerma, Agustí Barnadas

**Affiliations:** ^1^Institut d’Investigació Biomèdica Sant Pau, Barcelona, Spain; ^2^Hospital de la Santa Creu i Sant Pau, Barcelona, Spain; ^3^Department of Medicine, Universitat Autònoma de Barcelona, Bellaterra (Cerdanyola del Vallès), Spain; ^4^Centro de Investigación Biomédica en Red Cáncer, Madrid, Spain

**Keywords:** axillary lymph node dissection, breast cancer, sentinel lymph node, metastasis, microRNAs

## Abstract

Evidence that microRNAs (miRNAs) regulate the various steps of metastasis is increasing. Several studies have looked at the miRNA expression profile in primary breast tumors but few have compared primary tumor and sentinel lymph node (SLN) metastasis. We correlated the expression of miRNAs with the SLN status and the outcome of axillary lymph node dissection (ALND) in 60 patients with early breast cancer. We profiled the expression of miRNAs in paired breast tumor samples and SLNs using the NextSeq500 Illumina platform and key findings were validated by qPCR. MultiMiR Bioconductor and Reactome pathways analysis were performed to identify target genes and signaling pathways affected by altered expressed miRNAs. Our results show that nine miRNAs were differentially expressed in tumor tissues (*q* ≤ 0.05). In tumor samples, a 13.5-fold up-regulation of miR-7641-2 (*q* < 0.001) and a 2.9-fold down-regulation of miR-1291 (*q* < 0.001) were associated with tumors with positive SLNs. However, only down-regulation of miR-1291 (*q* = 0.048) remained significant in paired SLNs samples. Interestingly, a 10.5 up-regulation of miR-1291 in SLNs samples was associated with additional axillary lymph node involvement (*q* < 0.001). The enrichment analyses showed that canonical and non-canonical WNT pathways and negative regulation of various receptor tyrosine kinases signaling pathways were targets of miR-1291 and supports the role of miR-1291 as a tumor suppressor gene (TSG). Further studies are warranted to investigate the use of miR-1291 as a surrogate biomarker of SLN node metastasis in patients with early-stage breast cancer.

## Introduction

MicroRNAs (miRNAs) are a small (19–25 nt) non-coding RNA (ncRNA), expressed in a wide variety of organisms and highly conserved across species. MiRNAs regulate the expression of target genes by binding to complementary regions of messenger transcripts to repress their translation or regulate their degradation. The number of human miRNAs is currently over 1900 and the number of predicted target genes are in the range of thousands, with some estimates indicating that miRNAs target over 30% of the human genome. The overall emerging picture is that of a complex regulation level of gene expression, in which a single miRNA may control hundreds of targets (Condrat et al., 2020).

Several studies have shown that the expression signatures of miRNAs in human cancers are distinct from those in normal tissues (Janssen et al., 2010). In breast cancer, various studies have revealed a deregulation of miRNAs with clusters of miRNAs frequently being either over-expressed or down-regulated (Nana-Sinkam and Croce, 2011; Condrat et al., 2020). In addition, many cellular pathways are affected by the regulatory function of miRNAs and several human pathologies including cancers, have been associated with deregulation of the miRNAs (Dai and Ahmed, 2011) and play a pivotal role in various steps of the metastatic process (Nicoloso et al., 2009; Valastyan, 2012). Furthermore, analysis of miRNA expression correlated these with various clinicopathological factors (Blenkiron et al., 2007). However, few studies have compared primary tumor and lymph node (LN) metastasis (Smeets et al., 2011; Gravgaard et al., 2012; Cascione et al., 2013). Gravgaard et al. (2012) found a differential expression of miRNA-200 family and miRNA-9 in LN associated with the metastatic process. Cascione et al. (2013) reported analysis of 173 formalin-fixed paraffin embedded (FFPE) tumors and 53 matched LN in TNBC. They found two miRNA signatures that were independent predictors for overall survival (OS) and distant-disease free survival, respectively. In another study, Smeets et al. (2011) identified eight with measurable differences in gene and miRNA expression between N0 and N+ patients, suggesting that LN involvement is not a genetically random process.

Herein, we studied the miRNA expression profile in paired tumor and sentinel lymph node (SLN) from patients with early breast cancer. We used the one-step nucleic amplification (OSNA) assay (Tsujimoto et al., 2007) to accurately measure total metastatic volume in the SLN (Cserni, 2012), as an alternative to intraoperative microscopy-based pathological assessment of the SLN. The OSNA assay is a rapid molecular detection of SLN metastasis based on the semi-quantification of cytokeratin 19 (CK19) mRNA copy numbers (Tsujimoto et al., 2007). The CK19 mRNA copy number, also defined as total tumor load (TTL), has been shown to be a statistically significant parameter in predicting the risks of further positive axillary LN (aLN). Thus, only patients diagnosed with more than two macrometastatic SLN are further treated with axillary lymph node dissection (ALND) (Peg et al., 2013), the golden standard procedure for invasive breast cancer. The aLN status is the most powerful prognostic factor in breast cancer and knowledge of this is essential for making decisions about adjuvant therapy (Shek and Godolphin, 1988). However, ALND has been questioned in recent years because of inherent morbidity following the procedure without directly contributing to survival (Giuliano et al., 2010; Jagsi et al., 2014).

In this study, we investigated the potential use of miRNAs as surrogate biomarkers for the presence of metastases in the SLN and the outcome of ALND, in patients with early-stage breast cancer. We found a differential expression of various miRNAs associated with both the metastatic status of the SLN and the occurrence of further aLN metastases. This study provides a new framework to study the role of miRNAs in the regulation of tumor metastases and their impact on patient outcome.

## Materials and Methods

### Ethics Approval, Consent to Participate and Institutional Safety Procedures

This study was conducted according to the Declaration of Helsinki principles, with approval from the Clinical Research Ethics Committee at “Institut d’Investigacions Biomèdiques Sant Pau” (IIB Sant Pau). Written informed consent was obtained from all patients under institutional review board-approved protocols. All methods were performed in accordance with the relevant guidelines and regulations.

### Patient and Study Samples

This study included 60 patients with early-stage breast cancer treated with surgery. None of the patients had prior treatment with chemotherapy or radiation. Patients were chosen on the basis of paired biological samples availability and confirmed diagnose based on the histopathology of primary tumors and OSNA-diagnosed SLNs. Information about completion of ALND was available for all patients. Samples included hormone receptor (HR) positive [estrogen receptor (ER) or progesterone receptor (PR) positive], HER2 positive and triple negative (TN) tumors. We collected clinical and pathological parameters and clinical follow-up. Tumor stage was determined according to the AJCC/UICC system (Webber et al., 2014) and histological grade was determined using the Elston–Ellis grading system (Elston and Ellis, 1991). We collected paired tumor and SLNs for all 60 patients (*n* = 120 samples). All samples were classified according to the SLN status as negative (*n* = 20) or positive (*n* = 40). Positive samples were sub-classified as macrometastatic (*n* = 20) or micrometastatic (*n* = 20) (Tsujimoto et al., 2007).

### Sample Processing

Primary tumor samples were collected and processed within 30 min after surgery. A portion of each tumor was rapidly embedded in Tissue-Tek OCT (Sakura Finetek, Netherlands) and frozen using a histobath (Thermo Fisher Scientific, Waltham, MA, United States). Frozen tumor blocks were thin-sectioned and stained with hematoxylin/eosin and only those that were judged to contain at least 70% viable tumor by area were carried on for RNA extraction. Intraoperative SLN were evaluated by OSNA assay (Tsujimoto et al., 2007). The entire LN was homogenized in 4 mL of a lysis solution (Sysmex, Spain) and centrifuged at 10,000 × *g* at room temperature. A 2 μL sample of the supernatant was analyzed with the RD-100i system (Sysmex) and the remaining SLN homogenate was stored immediately at −80C.

### RNA Isolation

Total RNA was purified using TRIzol (Invitrogen) and mirVana (Ambion) reagents according to manufacturer’s instructions with some modifications. Briefly, Trizol homogenates were mixed with 1/10 volume of mirVana homogenizing solution to retain low molecular weight RNA species. The clear fraction containing total RNA was then purified using the mirVana miRNA isolation kit, following manufacturer’s instructions. Total RNA was eluted in 100 μl nuclease-free water.

### RNA-Sequencing

Total RNA concentration and purity was measured using the ribogreen RNA assay kit (Life Technologies) and the integrity was visualized with a TapeStation Bioanalyzer (Agilent Technologies, Inc.). Next, RNA pools were precipitated overnight with 2× volumes of absolute ethanol and 0.1× volume of 0.3 M sodium acetate at −80°C for cDNA library construction. Double-stranded cDNA libraries were constructed using the NEBNext Small RNA library Prep Set for Illumina (New England Biolabs, Ipswich, MA, United States) following manufacturer’s instructions. A quality check (QC) and size selection of the PCR amplified cDNA construct was performed using 6% polyacrylamide gel. Two biological replicates for each developmental stage were separately sequenced by the MiSeq (Illumina, San Diego, CA, United States) platform using sequenced runs of 2 × 75 paired-end reads and 1000× coverage to ensure proper quantification of the miRNA expression. A total of 59 tumors and 58 SLNs (117 paired-end, 2 × 75) sequences were successfully sequenced.

### Genome Annotation and Quantification of MiRNAs

Paired-end (forward-reverse) sample merging and initial bioinformatics analysis were performed with the CLCBio Genomics Workbench^®^ version 8.0.2 (Qiagen, Germany). A total of 234 fastq input files were generated and used in the analyses. The CLCBio software was used to align and map the trimmed reads to the human and mouse miRBases (version 19) and the Homo_sapiens.GRCh37.57 tracks from Ensembl. Up to two mismatches were allowed on the sequences. Mapping options were set as the program’s default. Count tables were generated with R programming language (R Development Core Team, 2011) and the EdgeR package (Bioconductor repository) (Robinson et al., 2010), using non-specific filtering for sequences having a reads-per-million value higher than 0, in at least half of the samples included on each experimental comparison. Transcript per million (TPM) was used as a normalization procedure to correct for differences in sequencing depth and to quantified RNA species.

### Differential Expression Analysis

Differential expression analyses were performed using the trimmed mean of M-values normalization method (TMM) (Robinson and Oshlack, 2010), based on the log-fold and absolute gene-wise changes in expression between samples. Differential expression analysis was performed using the EdgeR statistical software package (Bioconductor^1^). Principal component analysis (PCA) was performed using R programming and TMM-normalized quantifications from defined collections of samples as input. Volcano plots were constructed plotting the *p*-value (−log10) on the *y*-axis and the expression fold change (log2) between the two experimental groups on the *x*-axis. Wherever indicated, we have used fold regulation throughout the text to represent positive FC values as up-regulation (fold regulation is equal to 2^FC^) and negative FC values to indicate a down-regulation (fold regulation is equal to 2^–FC^).

### Quantitative Real-Time RT–PCR Validation Analysis

Selected miRNAs were validated by quantitative real-time RT–PCR (qPCR) in an ABI Prism 7500 Sequence Detection System using specific LNA PCR primers (Exiqon). The cDNA was constructed using the miRCURY LNA^TM^ Universal RT cDNA Synthesis Kit (Exiqon), diluted 40× and assayed in 10 μl PCR reactions according to manufacturer’s instructions. Each qPCR was assayed in triplicates and a no-template control (NTC) of water was purified and profiled like the rest of the samples. Analysis of the data was performed using the relative miRNA expression according to the comparative Ct (ΔΔCt) method using negative metastatic samples as reference. We used the geNorm (Andersen et al., 2004) or the Normfinder algorithm (Vandesompele et al., 2002) to select the best combination of two reference genes based on our qPCR data. Data from multiples plates were normalized using UniSp3 spike-in as interplate calibrators.

### Gene Targets Prediction and Enrichment Analysis of Gene Targets

The multiMiR Bioconductor’s package (Ru et al., 2014) was used to retrieve miRNA-target interactions from 14 external databases^2^ and the Reactome pathway database (Jassal et al., 2020) was use to performed enrichment analysis of target pathways and genes.^3^

### Statistics

Differentially expressed miRNAs obtained by next generation sequencing (NGS) were detected by an exact test based on conditional maximum likelihood (CML) included in the R Bioconductor package edgeR (Robinson et al., 2010). *p*-Values from NGS were corrected (*q*-values) for multiple testing using the Benjamini–Hochberg method (Benjamini and Hochberg, 1995). A *q*-value ≤ 0.05 was considered significant. In all group comparisons missing expression values were treated as zero. Differences in total numbers of miRNAs between groups were analyzed by two-sided parametric *t*-tests. The analysis of clinicopathological was performed using the Student’s *t*-test to compare quantitative variables, and the X2 or Fisher exact tests to compared qualitative variables. Disease free survival (DFS) was defined as the time from diagnosis to date of first relapse (local, regional, contralateral, or metastatic) or second primary cancer. OS was defined as the time from sample collection to death resulting from any cause. Patients lost to follow-up were censored at the last contact. Kaplan–Meier and log-rank analyses were used to compare DFS and OS. Differential expression by qPCR of selected miRNAs was analyzed using an independent sample *t*-test with a Levene’s test for equality of variances. The *p*-values were calculated using a Student’s *t*-test of the replicate 2^–ΔCT^ values for each miRNA in the different groups compared. A two-sided *p*-value ≤ 0.05 was considered significant.

## Results

### Patients Characteristics

Patient and tumor characteristics are summarized in Table 1. We have analyzed 60 patients for whom paired tumor and SLN samples were available. A total of 20 patients had negative SLNs (33%) and 40 patients had positive SLNs (67%). Of those patients with positive SLNs, 20 (33%) SLNs were diagnosed as macrometastasis and 20 (33%) SLNs as micrometastasis. All patients diagnosed with macrometastasis received ALND of whom 5 (25%) had further aLN involvement. Our study included 46 patients with HR-positive tumors (77%), 9 patients with HER-2 positive carcinomas (15%), and 5 patients with TN (8%) tumors.

**TABLE 1:** Basic patient and tumor characteristics.

Variable		Total (%)	LN negative (%)	LN positive (%)
***N***	*N* (%)	60 (100)	20 (100)	40 (100)
Age (years)	≤50	9 (15)	2 (10)	7 (18)
	>50	51 (85)	18 (90)	33 (82)
	Mean + SD	63.6 ± 14.2	68.8 ± 11.9	60.9 ± 14.7
	Median (range)	64 (26-88)	71 (47-87)	60 (26-88)
Tumor stage	I	18 (30)	12 (60)	6 (15)
	II	40 (67)	8 (40)	32 (80)
	III	2 (3)	0 (0)	2 (5)
Tumor status	T1	32 (53)	12 (60)	20 (50)
	T2	27 (45)	8 (40)	19 (48)
	T3	1 (2)	0 (0)	1 (2)
Tumor grade	1	3 (5)	1 (5)	2 (5)
	2	35 (58)	14 (70)	21 (52)
	3	22 (37)	5 (25)	17 (43)
Node status	N0	20 (33)	20 (100)	0 (0)
	N+	40 (67)	0 (0)	40 (100)
SLN OSNA diagnosis	Negative	20 (33)	20 (100)	0 (0)
	Micrometastasis	20 (33)	0 (0)	20 (50)
	Macrometastasis	20 (33)	0 (0)	20 (50)
aLN status*	Negative	15 (75)	0 (0)	15 (75)
	Positive	5 (25)	0 (0)	5 (25)
CK19 status**	Low	12 (60)	0 (0)	12 (60)
	High	8 (40)	0 (0)	8 (40)
Tumor type	Unifocal	36 (60)	13 (65)	23 (58)
	Multifocal	20 (33)	7 (35)	13 (32)
	Multicentric	4 (7)	0 (0)	4 (10)
ER status	Negative	6 (10)	2 (10)	4 (10)
	Positive	54 (90)	18 (90)	36 (90)
PR status	Negative	18 (30)	7 (35)	11 (28)
	Positive	42 (70)	13 (65)	29 (72)
Receptor status	HR-positive	46 (77)	16 (80)	30 (75)
	HER-2 positive	9 (15)	3 (15)	6 (15)
	TN	5 (8)	1 (5)	4 (10)
Ki67 status	>20%	14 (23)	4 (20)	10 (25)
	≤20%	46 (77)	16 (80)	30 (75)
Lymphovascular invasion	Negative	48 (80)	18 (90)	30 (75)
	Positive	12 (20)	2 (10)	10 (25)
Menopausal status	Premenopausal	10 (17)	2 (10)	8 (20)
	Postmenopausal	50 (83)	18 (90)	32 (80)
Breast affected	Left	41 (68)	12 (60)	29 (73)
	Right	19 (32)	8 (40)	11 (27)
Breast surgery	Mastectomy	19 (32)	5 (25)	14 (35)
	Lumpectomy	41 (68)	15 (75)	26 (65)

aLN, axillary lymph node; ER, estrogen receptor; LN, lymph node; PR, progesterone receptor; TN, triple negative. *CK19 mRNA copy number >15,000 in the subgroup of patients with macrometastatic SLNs. **Presence of additional aLN in the subgroup of patients with macrometastases.

### RNA-Sequencing

Prior to sequencing the samples were subjected to a QC. Three samples (one tumor and two SLNs) were excluded from further analyses because they did not pass all of the QC metrics, including the average read quality, the average base quality and the read length distribution with a *Q*-score > 30 (99.9% correct) (Cock et al., 2010). The remaining 59 tumors and 58 SLNs were successfully sequenced resulting in a total of 117 paired samples. All samples were sequenced in nine runs with a minimum and maximum read number of 0.33 and 34.5 million reads number, respectively. This resulted in a median 2.5 million read number per sample (Supplementary Table 1). Following sequence trimming, all reads containing identical insert sequences were collapsed into a single read, which were passed into the analysis pipeline. On average we obtained 0.92 million reads for each sample resulting in an average genome mapping rate of 26.9%. The remaining unmapped reads were usually from degraded RNAs that could not be uniquely mapped. After mapping and counting to relevant entries in mirbase_20 database, the number of known miRNAs was calculated using TPM to measure expression.

### Differential Expression

**FIGURE 1:**
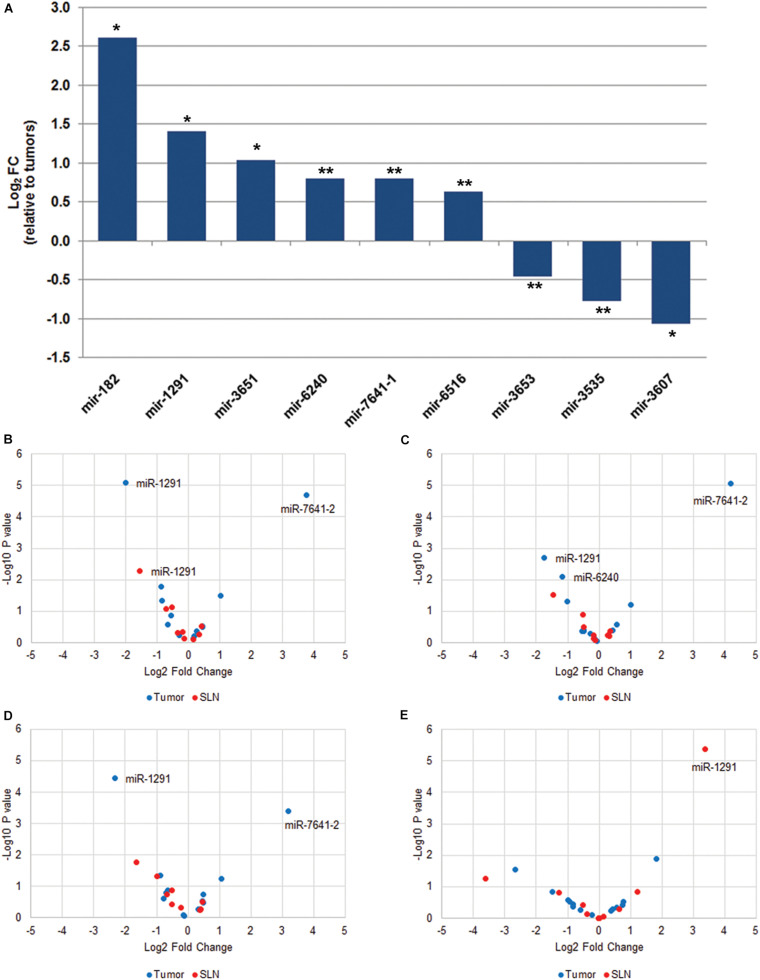
MiRNA expression profile in paired tumor and SLN samples from breast cancer patients. **(A)** Total number of miRNAs with significant differential expression (*q* < 0.05) in tumor samples compared to paired SLN samples. Data is expressed as log2 fold change relative to tumor samples. *q < 0.001, **q < 0.05. The volcano plots show differentially expressed miRNAs in tumor (blue) and SLN (red) samples according to the locoregional metastatic status: positive vs. negative **(B)**, macrometastasis vs. negative **(C)**, micrometastasis vs. negative **(D)**, and subgroup of patients with macrometastasis that were treated with ALND and grouped according to the aLN status (positive vs. negative) **(E)**. The data show the relationship between the *p*-values (*y*-axis) and the fold change (*x*-axis) between the experimental groups. Only miRNAs with *q* < 0.05 are shown in the plots.

To identify differentially expressed miRNAs between paired samples. First, we performed a data reduction analysis to compare the miRNA expression profile in paired tumor and SLN samples. Our results show that the two types of samples separated into two different groups, suggesting that the miRNA expression profile between paired tumor and SLNs samples from a same patient is different. Other prognostic factor such as tumor stage or tumor grade were in part responsible for grouping the tumor samples (Supplementary Figure 1). We observed significant differences in the expression of 9 miRNAs (*q* < 0.05) in tumors samples compared to SLNs. Six miRNAs were up-regulated (miR-182, miR-1291, miR-3651, miR-6240, miR-7641-1, and miR-6516) and three miRNAs were down-regulated (miR-3653, miR-3535, and miR-3607) (Figure 1A). We further validated the expression for all nine miRNAs using specific qPCR assays.

Next, we analyzed tumor and SLN samples independently in relation to the metastatic SLN status. We first compared negative (*n* = 20) vs. positive (*n* = 40) samples (Figure 1B and Supplementary Table 2). Our results show a 13.9-fold up-regulation of miR-7641-2 (*q* < 0.001) and a fourfold down-regulation of miR-1291 (*q* < 0.001) in positive tumor samples. A non-significant 1.9-fold down-regulation was also observed for miR-6240 (*q* = 0.076). In contrast, in positive SLNs samples only a 2.8-fold down-regulation of miR-1291 remained significant (*q* = 0.048). Next, we determined whether the differential expression of miRNAs observed in positive samples compared to negative samples was retained when samples were sub-classified as either macro- or micrometastasis. We observed a similar deregulation pattern in tumor samples. Macrometastatic tumors showed an 18.4-fold up-regulation (*q* < 0.001) of miR-7641-2, a 3.2-fold down-regulation of miR-1291 (*q* = 0.014) and a 2.3-fold down-regulation of miR-6240 (*q* = 0.038). In contrast, no differential expression was observed for SLNs diagnosed as macrometastasis (Figure 1C and Supplementary Table 2). Similar results were obtained when we compared micrometastatic and negative tumors or SLNs (Figure 1D and Supplementary Table 2). Interestingly, no significant differences were observed when comparing macrometastatic to micrometastatic tumor or SLNs (Supplementary Table 2), suggesting that the volume of the metastatic lesion do not translate in differences in the expression profile of miRNAs.

It has been reported that patients with T_1__–__2_N_0__–__1_ invasive non-metastatic breast cancer treated with breast conserving surgery and randomized to undergo ALND after SLN dissection vs. SLN dissection alone, showed no significant differences in local recurrence or regional recurrence (Giuliano et al., 2010; Jagsi et al., 2014). Therefore, we investigated the expression of miRNAs in tumors (*n* = 19) or SLNs (*n* = 20) from patients diagnosed as macrometastasis and with additional positive aLNs (*n* = 5). Our results show that in SLNs samples, a 10.6-fold up-regulation of miR-1291 (*q* < 0.001) was associated with additional aLN metastases (Figure 1E). In tumor samples, we found a differential expression of miR-182 (*p* = 0.013) and miR-7641-2 (*p* = 0.029) but none were significant after FDR correction (*q* > 0.05) (Figure 1E). We have further analyzed this subset of samples in relation to the TTL since it has been reported that a TTL cut-off value >15,000 copies of CK19 is associated with additional aLN metastasis (Peg et al., 2013). Our results show a negative correlation of miR-3535 and miR-3653 in tumors and a positive association of miR-1291in SLNs in relation to the TTL. However, none of these miRNAs passed the FDR correction (*q* > 0.05) (data not shown).

Our tumor samples included 46 HR-positive (77%), 9 HER2-positive (15%), and 5 TN (8%) tumors (Table 1). No differential expression of miRNAs was found between molecular subtypes in tumor samples (*n* = 59). However, in SLN samples, miR-1291 showed a 10-fold up-regulation in HER2-positive samples (*q* = 0.0046) compared to HR-positive SLNs and a 3.5-fold up-regulation of miR-3607 in HER2-positive SLNs compared to TN SLNs (*q* = 0.033) (Table 2). Next, we investigated the expression profile of miRNAs according to the molecular subtype and the SLN metastatic status. In TN tumor samples with positive SLNs, an 18.1 down-regulation of miR-3535 (*q* = 0.048) was observed compared with HR-positive tumors. A similar non-significant 20.2-fold down-regulation was observed when comparing TN with HER-2 positive tumors with positive LNs (*q* = 0.06). No differences were observed in SLN samples (data not shown).

**TABLE 2:** Differential expression of miRNAs in tumor samples (*n* = 59) and SLN (*n* = 58) according to the breast cancer molecular subtypes.

Sample	Comparison	miRNA	log2 FC	*p*-Value	*q*-Value	Fold regulation	
Tumor	HR+ vs. HER2+	mir-21	–1.28	0.012	0.175	2.4	Down-regulation
	HR+ vs. TNBC	mir-3651	1.75	0.053	0.596	3.4	Up-regulation
	HER2+ vs. TNBC	mir-3651	2.13	0.032	0.352	4.4	Up-regulation
		mir-21	1.88	0.050	0.352	3.7	Up-regulation
SLN	HR+ vs. HER2+	mir-1291	3.323	0.0005	0.005	10.0	Up-regulation
		mir-3607	–0.756	0.032	0.143	1.7	Down-regulation
	HR+ vs. TNBC	mir-3607	1.03	0.051	0.319	2.1	Up-regulation
		mir-6516	1.56	0.074	0.319	3.0	Up-regulation
	HER2+ vs. TNBC	mir-3607	1.79	0.004	0.033	3.5	Up-regulation
		mir-6516	2.27	0.017	0.075	4.9	Up-regulation
		mir-3653	1.09	0.044	0.132	2.1	Up-regulation
		mir-1291	–1.98	0.073	0.164	3.9	Down-regulation

*FC, fold change.*

### Gene Target Prediction and Enrichment Analyses

**FIGURE 2:**
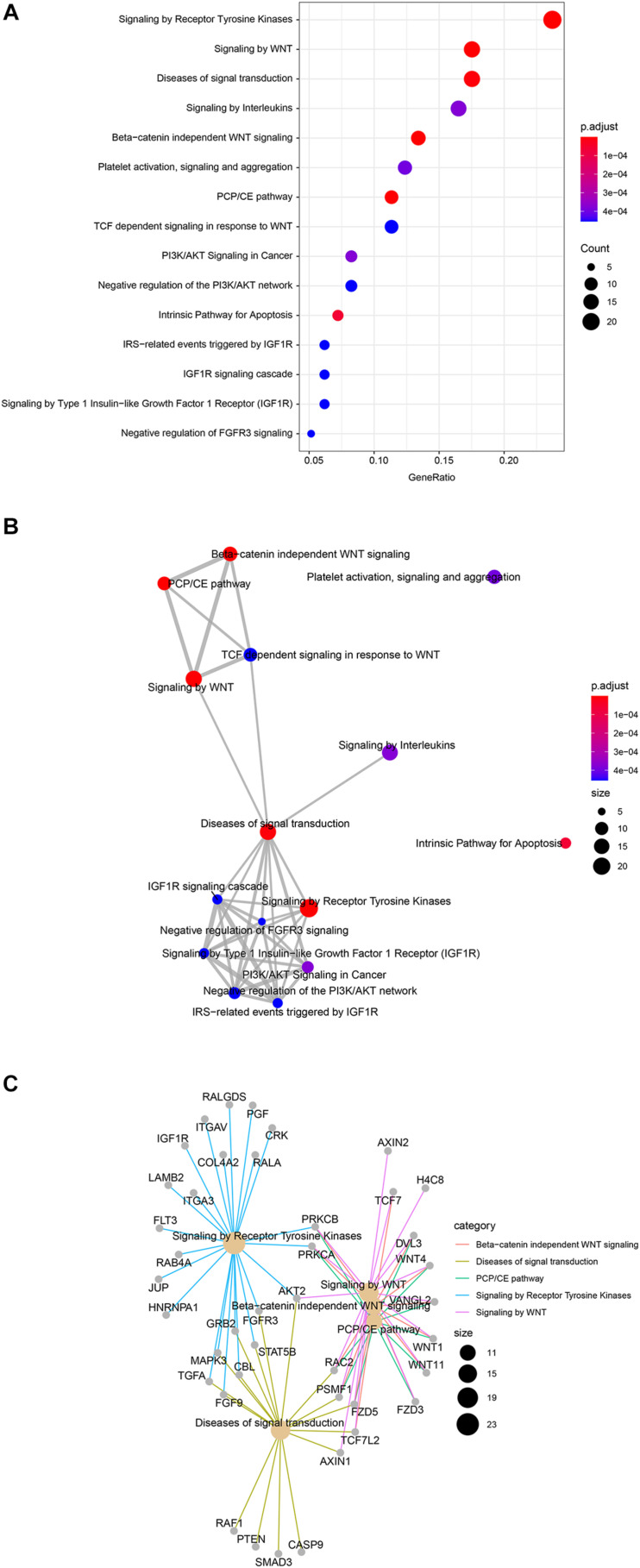
Reactome pathway enrichment in miR-1291 targeted genes. **(A)** Dot plot graph shows the 15 most significant enriched pathways and the ratio between the genes that belongs to that category and the total number of genes in the pathway. The color of the nodes indicates the *p*-value and the size of the nodes the number of genes in the data that belong to that pathway **(B)** Enrichment map of the top 15 signaling pathways grouped by similarity. Nodes are colored by *p*-value and their size reflects the number of genes found in that term. **(C)** Network plot of the genes found in the top five enriched signaling pathways showing the linkages between genes and pathways. The size of nodes indicates the number of genes found in that pathway.

To investigate target genes of miR-1291 we used the multiMiR Bioconductor’s package to retrieve validated targets from mirecords, mirtarbase, and tarbase databases and predicted targets from diana-microt, elmmo, microcosm, miranda, mirdb, pictar, pita, and targetscan databases. A total of 171 validated targets were found (Supplementary Table 3). To investigate the pathways and genes associated with miR-1291 we used the Reactome pathway database. The analyses show the most relevant pathways and the number of significant target genes within each category (Figure 2A and Supplementary Table 4), the interactions between these pathways (Figure 2B) and the targets genes associated with these pathways and how these genes and signaling pathways are linked (Figure 2C). Overall, our results show that the top five significant pathways regulated by miR-1291 are signaling by WNT, planar cell polarity/convergent extension (PCP/CE) pathway, β-catenin independent WNT signaling, diseases of signal transduction and signaling by receptor tyrosine kinases (RTKs). Interestingly, all target genes within these categories (Figure 2C) were included in the validated mirtabase and tabase databases (Supplementary Table 3).

### Clinical Status

**FIGURE 3:**
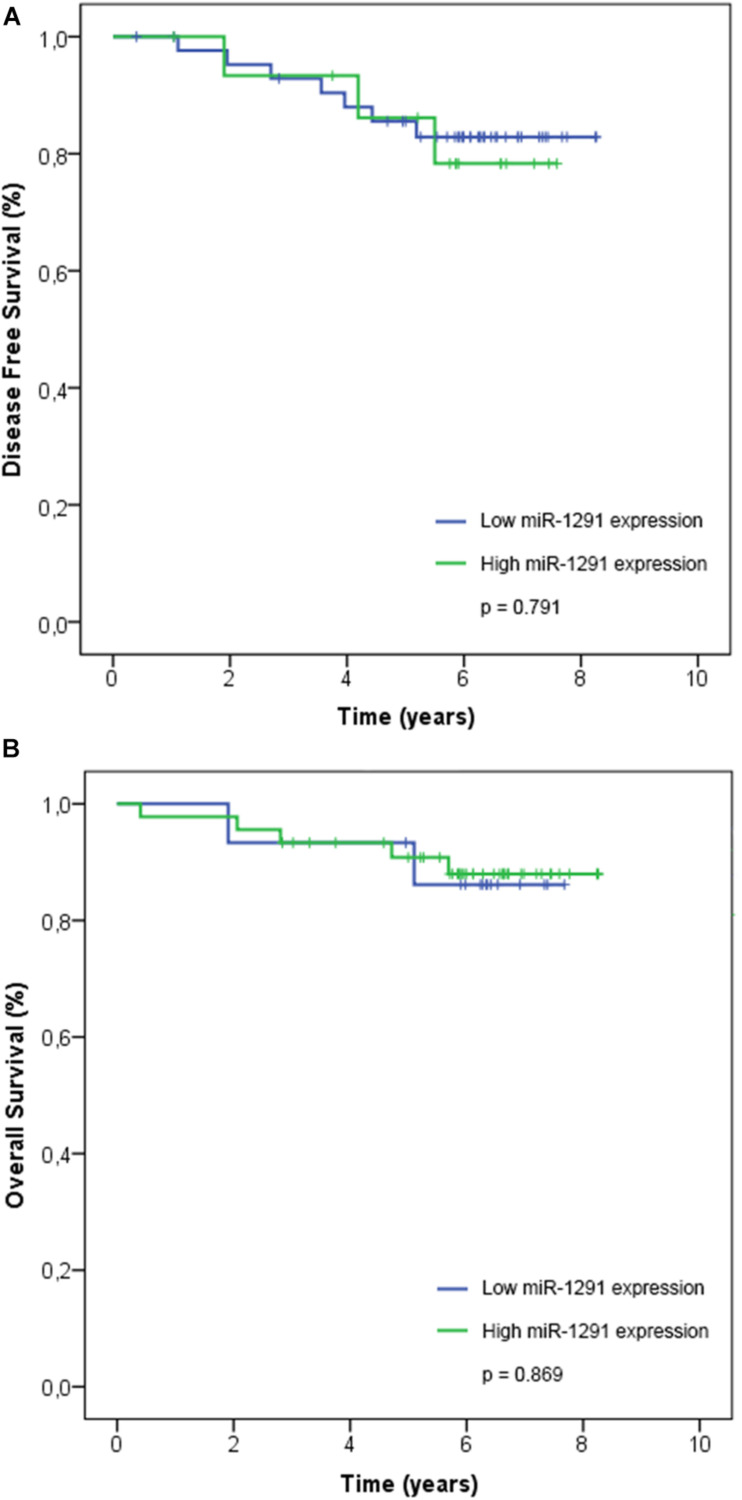
Association of miR-1291 expression with patient outcome. The expression levels of log2-transformed miR-1291 was correlated with **(A)** disease-free survival and **(B)** overall survival. Kaplan–Meier curves and log-rank tests are shown.

Our series include 60 patients with early breast cancer and we reported recurrence in 10/60 (17%) patients. Four patients had locoregional recurrences (breast) and six patients had distant metastasis (two cases in the liver and one case in the lung, pancreas, bone, or brain). At last follow-up, three patients with recurrences in their breast were reported alive without any evidence of disease, whereas 5/10 other recurrences (breast, lung, bone, and pancreas) were alive with disease. We reported seven deaths in our series, three deaths were due to complications related to the disease (two deaths were related to liver metastases and one death related to lung metastases), and four deaths non-related to disease (general deterioration related to age and/or associated comorbidity). We performed a survival analysis using the expression of miR-1291. The Kaplan–Meier and log-rank analyses showed that the expression levels of miR-1291 (high vs. low expression) were not significantly associated with DFS nor OS (Figure 3).

## Discussion

In this study we have correlated the expression of miRNAs with SLN metastatic status and the outcome of ALND. We profiled the miRNA expression using NGS and key findings were validated by qPCR. We analyzed samples from 60 patients with early breast cancer for whom paired primary tumor and SLNs were available. Overall, we found a poor correlation in the miRNA expression profile from SLNs compared to match primary tumors samples from the same patient. These differences are likely due to histological differences between the LN and the tumor. Whereas the SLN is constituted mainly by lymphoid and monocytes cells the tumor tissue is formed mostly by epithelial and mesenchymal cells. Nonetheless, we found nine differentially expressed miRNAs in tumor tissues compared to SLN (*q* ≤ 0.05). Of those, only four miRNAs showed a fold de-regulation ≥2.0 (either up-regulation or down-regulation). The two most de-regulated miRNAs were miR-182 (6.2-fold up-regulation, *p* < 0.001) and miR-3607 (2.1-fold down-regulation, *p* < 0.001). Our data agrees with a previous report showing that miR-182 directly targets MIM (Missing in Metastasis), which suppresses metastasis by inhibiting Ras homolog family member A (RhoA) activity and stress fiber formation in breast cancer cells (Lei et al., 2014). In addition, miR-182 expression was reported to be higher in primary tumors with positive SLNs metastasis and correlated with earlier relapse-free and metastasis-free survivals (Lei et al., 2014). On the other hand, miR-3607 has been shown to be widely attenuated in prostate cancer (PCa) patients and low expression levels were significantly associated with higher PCa stage, Gleason score, serum PSA levels, tumor progression, and poor survival outcome (Saini et al., 2014).

Given that paired tumor and SLN samples in our study showed different miRNA expression profiles, we analyzed both types of samples independently in relation to the locoregional metastatic status. We show that down-regulation of miR-1291 in both tumor and SLN samples was associated with positive SLN metastases. Interestingly, we found that in the subgroup of positive patients that underwent ALND and had further aLN metastases the expression of miR-1291 was significantly higher compare to patient with negative aLNs. However, these results must be interpreted with caution since only five patients fulfilled those characteristics in our dataset and thus, further clinical validation in a larger cohort of patients will be required to establish these observations. If confirmed, these results would suggest that the degree of down-regulation might have an effect on how miR-1291 modulates its target genes and affect various signaling pathways. Nonetheless, our study did not correlate the miRNA expression with the transcriptional expression in the same samples. Therefore we could not determine if miR-1291 function as an oncogene as it has been described for hepatocellular carcinomas and liver cirrhosis patients (Hagag et al., 2020) or in microvesicles derived from medulloblastoma cell lines with stem-like properties (Kaid et al., 2020). Nevertheless, our data suggest the contrary and agrees with previous studies showing that the expression of miR-1291 is significantly down-regulated in various human cancers, including pancreatic cancer (Bi et al., 2014; Tu et al., 2016) esophageal squamous cell carcinoma (ESCC) (Luo et al., 2015), PCa (Cai et al., 2019) and renal cell carcinoma (RCC) (Yamasaki et al., 2013). In these studies, loss of tumor-suppressive miR-1291 have been reported to enhanced cell proliferation, migration, and invasion through up-regulation of various miR-1291 targets, whereas restoration of miR-1291 expression in cancer cell lines and animal models reverted those effects. For example, miR-1291 restoration repressed tumorigenesis in prostate and pancreatic xenograft tumor models via inhibition of Mediator of RNA polymerase II transcription subunit 1 (MED1) (Cai et al., 2019), N-methylnicotinamide (NMN) (Bi et al., 2014). In addition, miR-1291 reduced the protein levels of target genes including ATP Binding Cassette Subfamily C Member 11 (ABCC1), Forkhead box protein A2 (FOXA2), Anterior Gradient 2 (AGR2), methyl CpG binding protein 2 (MeCP2) and carnitine palmitoyltransferase 1C (CPT1C) resulting in the suppression of growth and tumorigenesis of human breast and pancreatic cell lines (Bi et al., 2014; Li et al., 2015; Tu et al., 2016; Chen et al., 2020). Similar effects have been reported in RCC through targeting SLC2A1/GLUT1 (Yamasaki et al., 2013) which it has also been reported to be overexpressed in human breast carcinomas (da Cunha et al., 2013), in esophageal carcinomas by inhibiting mucin 1 (MUC1) (Luo et al., 2015), whereas miR-1291 acts upstream of the Rho GTPase-activating protein 29 (ArhGAP29) to negatively regulate the RhoA/ROCK1 epithelial mesenchymal transition (EMT) pathway, ultimately leading to endometrial fibrosis (Xu et al., 2017). Interestingly, our target prediction analysis identified the reported genes as miR-1291 targets, whereas our enrichment analysis pointed out various signaling pathways and several miR-1291 target genes that are commonly overexpressed in breast cancer, suggesting that in fact miR-1291 acts as a tumor suppressor gene (TSG). For instance, our data shows that miR-1291 targets genes that are involved in both canonical and non-canonical WNT signaling and regulate many aspects of cell polarity, morphogenesis, and development (Saito-Diaz et al., 2013). In some contexts, both the canonical and non-canonical WNT signaling contribute to tumor formation by promoting cell migration, invasiveness, and metastasis (van Amerongen, 2012; VanderVorst et al., 2019). One of the non-canonical WNT pathways we identified in relation to miR-1291 is the PCP pathway, which contains core Wnt/PCP components that are overexpressed in a variety of solid tumors and have been directly implicated in promoting tumor cell migration and metastasis (VanderVorst et al., 2018). Our data shows that two of the Wnt/PCP genes (WNT11 and VANGL2) are targets of miR-1291 and both have been reported to promote cell motility and metastasis in breast cancer (Luga et al., 2012). The Frizzled-5 (FDZ5) and FDZ3 are another non-canonical WNT members targeted by miR-1291 that bind to the FZD receptor and leads to activation of small Rho GTPases (RAC2) and JNK, which regulate the cytoskeleton and coordinate cell migration and polarity (Schlessinger et al., 2009). Overexpression of FDZ5 and FDZ3 has been associated with cancer aggressiveness in human breast carcinomas (da Cunha et al., 2013; Kazanietz and Caloca, 2017). Furthermore, we showed that miR-1291 is associated with negative regulation of various RTKs pathways, such as the PI3K/AKT, IGFR1, and FGFR3 signaling pathways that have a pivotal role in the regulation of cancer proliferation, angiogenesis, and metastasis in breast cancer (Butti et al., 2018). We have identified several well-known oncogenes that participate in these pathways, including MAPK3, RAF1, AKT2, or TGFA as validated targets of miR-1291. Collectively, our data supports the role of miR-1291 as a TSG in the onset of locoregional metastasis in early breast cancer patients. Further research is warranted to investigate the expression of miR-1291 as a potential surrogate marker of SLN involvement in early breast cancer patients.

Our results complement other studies that have compared the miRNA expression profile between primary tumor and LNs from the same patients (Smeets et al., 2011; Gravgaard et al., 2012; Cascione et al., 2013). Gravgaard et al. (2012) found a differential expression of miRNA-200 family and miRNA-9 in LN associated with the metastatic process. Cascione et al. (2013) reported the analysis of 173 FFPE tumors and 53 matched LN in TNBC. They found two miRNA signatures (miR-16, 155, 125b, 374a and miR-16, 125b, 374a, 374b, 421, 655, 497) that were independent predictors for OS and distant-DFS, respectively. In another study, Smeets et al. (2011) identified eight miRNAs (miR-195, miR-191, miR-132, miR-203, miR-431, miR-16, miR-30c, miR-30a) with measurable differences in gene and miRNA expression between N0 and N+ patients. However, the deregulated miRNAs described here is different from previously published (Smeets et al., 2011; Gravgaard et al., 2012; Cascione et al., 2013). Various reasons might explain this discrepancy. First, the different molecular breast cancer subtypes investigated in each study. Our cohort of patients comprised mostly luminal tumors, whereas others analyzed only TNs tumors (Cascione et al., 2013) or a mix of molecular subtypes (Smeets et al., 2011; Gravgaard et al., 2012). Second, the type of starting biological material used in previous studies, which was either archive FFPE specimens (Gravgaard et al., 2012; Cascione et al., 2013) or frozen sections (Smeets et al., 2011). In contrast, we used the entire fresh SLN homogenate employed in the OSNA assay (Cserni, 2012). The OSNA assay was developed as an alternative to intra-operative microscopy-based pathological assessment of the SLN because such methods (frozen sections, touch imprints scrapes, or a combination of these) have a limited ability to accurately measure total metastatic volume in a LN (Cserni, 2012). Thus, our study was not limit by sampling bias that might have occurred from using different parts of the LN. Moreover, the OSNA technique allows for the immediate sample processing required to preserve RNA for research purposes. Therefore, we did not encounter the limitations of using RNA extracted from archival FFPE tissues, which has often suffered chemical modification, cross-linking, and degradation over time as a result of the fixation and archiving methods (Esteve-Codina et al., 2017). Finally, a recent study has shown that differences on the protocol used for the preparation of the NGS libraries results in over- or underrepresentation of miRNAs in the sequencing library (Coenen-Stass et al., 2018). The use of different NGS library preparation kits and protocols could result on an inefficient size-selection or in an enrichment of other RNA species therefore decreasing the number of usable miRNAs reads. That could be our case, given that the library kit used to generate our RNA-seq data (NEBNext) has been recently reported to produce the least mirRNA with the greatest coefficient of variation (Coenen-Stass et al., 2018). In fact, a preliminary analysis of our RNA-seq data in tumor samples shows an enrichment in small nucleolar RNAs (snoRNAs) (data not published). Interestingly, several of the miRNAs described in this study appear to be derived from snoRNAs small ncRNAs (Thorenoor and Slaby, 2015). Other studies have reported human snoRNAs fragments that are annotated as miRNAs, including miR-1291 (SNORA2C), miR-1259 (SNORD12B), miR-1248 (SNORA81), miR-3535, and miR3653 (SNORD125), all of which are reported in our study. Similar reports have described other human snoRNAs showed miRNA-like processing signatures that can inhibit the expression of targets genes (Ender et al., 2008; Brameier et al., 2011; Makarova et al., 2013; Thorenoor and Slaby, 2015). Further studies are warranted to understand the role of snoRNAs in the mechanisms of invasion in breast cancer.

## Conclusion

To summarize, in this study we have analyzed the miRNA expression profile in paired tumor and SLN from patients with early breast cancer. We found that miR-1291 is differentially expressed in breast cancer patients with positive SLN and further involvement of aLN metastases. Our prediction and enrichment analyses provides a comprehensive understanding of the involvement of miR-1291 in promoting metastasis. Altogether, our data supports the role of miR-1291 as a TSG. Given the increasing importance of miRNAs in regulating cellular processes and the clinical success of the OSNA assay in detecting LN metastases, further research is warranted to investigate the direct implications of our results (Condrat et al., 2020) in treatment decision-making and (Janssen et al., 2010) in developing more effective blood-based clinical tests.

## Data Availability Statement

The original contributions presented in the study are publicly available. The datasets can be found in the Sequence Research Archive under ID PRJNA663033 and are available for download here http://www.ncbi.nlm.nih.gov/bioproject/663033.

## Ethics Statement

The studies involving human participants were reviewed and approved by the Comité Ètic d’Investigació Clínica (CEIC) Hospital de la Santa Creu i Sant Pau Sant Quintí 77-79, 08041 Barcelona, Catalunya, Spain. The patients/participants provided their written informed consent to participate in this study.

## Author Contributions

DE, LL-V, and AB were involved in the conceptualization of the project. DE, LL-V, OB, JM, JP, AM, CA, TR, and EL were involved in resources, investigation, and methodology. DE, LL-V, EL, and AB were involved in analysis and interpretation of data. All authors have contributed to the writing of the manuscript and have critically reviewed it.

## Conflict of Interest

The authors declare that the research was conducted in the absence of any commercial or financial relationships that could be construed as a potential conflict of interest.

## Footnotes

1 http://www.bioconductor.org/

2 http://multimir.ucdenver.edu

3 https://reactome.org/
