# Supplementary figures and images for: MicroRNA-1291 Is Associated With Locoregional Metastases in Patients With Early-Stage Breast Cancer
Source: Front Genet. 2020 Dec 2;11:562114. doi: 10.3389/fgene.2020.562114 (PMC7738477; doi:10.3389/fgene.2020.562114)

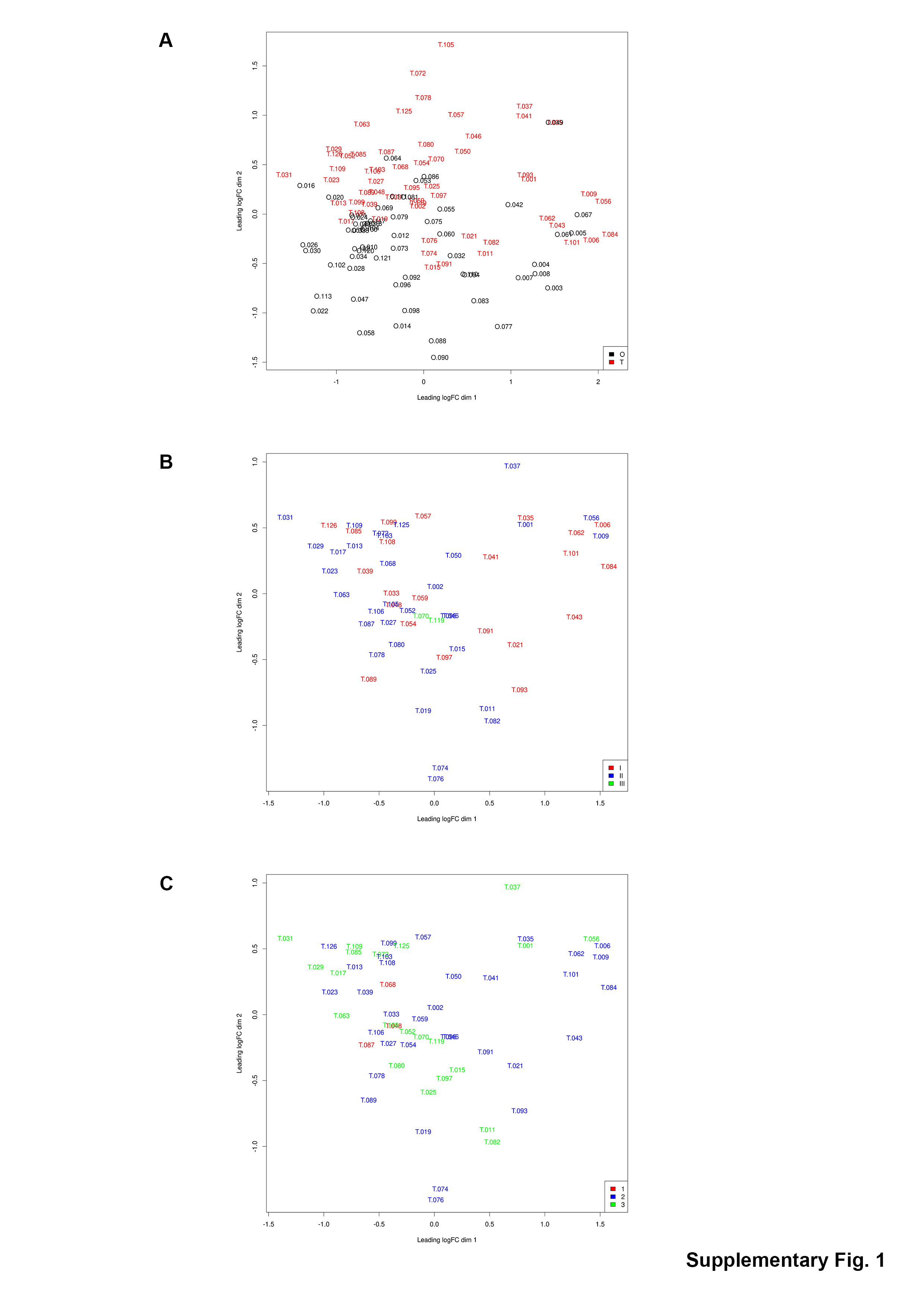

Supplement: Supplementary file 2 [file Image_1.TIF]
